# Hypothermia Alleviates Reductive Stress, a Root Cause of Ischemia Reperfusion Injury

**DOI:** 10.3390/ijms231710108

**Published:** 2022-09-03

**Authors:** Kattri-Liis Eskla, Hans Vellama, Liisi Tarve, Hillar Eichelmann, Toomas Jagomäe, Rando Porosk, Vello Oja, Heikko Rämma, Nadežda Peet, Agu Laisk, Vallo Volke, Eero Vasar, Hendrik Luuk

**Affiliations:** 1 Institute of Biomedicine and Translational Medicine, Department of Physiology, University of Tartu, 50412 Tartu, Estonia; 2 Center of Excellence for Genomics and Translational Medicine, University of Tartu, 50412 Tartu, Estonia; 3 Institute of Biomedicine and Translational Medicine, Department of Pathophysiology, University of Tartu, 50412 Tartu, Estonia; 4 Institute of Technology, Faculty of Science and Technology, University of Tartu, 50412 Tartu, Estonia; 5 Institute of Biomedicine and Translational Medicine, Department of Biochemistry, University of Tartu, 50412 Tartu, Estonia

**Keywords:** hypothermia, organ transplant, ischemia reperfusion injury, reductive stress, cellular respiration, hypoxia

## Abstract

Ischemia reperfusion injury is common in transplantation. Previous studies have shown that cooling can protect against hypoxic injury. To date, the protective effects of hypothermia have been largely associated with metabolic suppression. Since kidney transplantation is one of the most common organ transplant surgeries, we used human-derived renal proximal tubular cells (HKC8 cell line) as a model of normal renal cells. We performed a temperature titration curve from 37 °C to 22 °C and evaluated cellular respiration and molecular mechanisms that can counteract the build-up of reducing equivalents in hypoxic conditions. We show that the protective effects of hypothermia are likely to stem both from metabolic suppression (inhibitory component) and augmentation of stress tolerance (activating component), with the highest overlap between activating and suppressing mechanisms emerging in the window of mild hypothermia (32 °C). Hypothermia decreased hypoxia-induced rise in the extracellular lactate:pyruvate ratio, increased ATP/ADP ratio and mitochondrial content, normalized lipid content, and improved the recovery of respiration after anoxia. Importantly, it was observed that in contrast to mild hypothermia, moderate and deep hypothermia interfere with HIF1 (hypoxia inducible factor 1)-dependent HRE (hypoxia response element) induction in hypoxia. This work also demonstrates that hypothermia alleviates reductive stress, a conceptually novel and largely overlooked phenomenon at the root of ischemia reperfusion injury.

## 1. Introduction

Ischemia reperfusion injury is a pathological process that is the most common cause of organ transplant rejection/failure. Cooling has proven to be the first line of defense against hypoxic injury. At the cellular level, the fundamental justification of cooling rests on the premise that a decrease in temperature reduces cellular metabolism and the requirements for oxygen [1], however, cold ischemia can only attenuate cellular injury. The two primary options for organ preservation are static cold storage (SCS) and machine perfusion (MP) [1,2,3]. MP settings range from hypothermic (1–18 °C) [3,4,5,6], subnormothermic (20–34 °C) [7,8], to normothermic (35–38 °C) [6,9]. Acceptable cold ischemic times for SCS and MP range from 4 to 44 h [10,11,12,13,14,15,16,17]. To date, SCS at 4 °C has remained the most common preservation technique [18]. However, there is increasing evidence that MP may result in better outcomes after transplantation compared to SCS [2,19]. Although SCS is simple, effective, and cost-efficient [2], increasing cold ischemic time (CIT) triggers a cascade of noxious effects, which contributes to ischemia reperfusion injury, graft dysfunction, and failure [20]. A number of studies have looked at the molecular events associated with cooling. For instance, it affects pathways leading to apoptosis, inflammation, and free radical production, leads to activation of antioxidant system, suppression of dopamine and glutamate release, prevention of proteases responsible for degrading the extracellular matrix, expression of BDNF (brain-derived neurotrophic factor), GDNF (glial cell line-derived neurotrophic factor), and other neurotrophins [21,22,23,24]. However, protective effects of cooling are still largely attributed to the fact that lower temperature reduces metabolic demand and slows down cellular processes [25].

Limited oxygen availability during ischemic/hypoxic conditions disturbs the dynamic balance between citric acid cycle (CAC) and electron transport chain (ETC), leading to a pathological state and potentially permanent tissue damage as exemplified by the ischemia reperfusion injury [26]. Recent evidence suggests that markedly increased reducing power (i.e., the accumulation of reducing equivalents such as NADH (nicotinamide adenine dinucleotide hydrogen), NADPH (nicotinamide adenine dinucleotide phosphate hydrogen), succinate, etc.) is the primary cause of pathology in ischemia reperfusion injury [26]. The paradigm of reductive stress stems from the understanding that citric acid cycle converts nutrients (e.g., pyruvate, glutamate, and fatty acids) to reducing equivalents. In the mitochondria, reducing equivalents (e.g., NADH, NADPH, succinate) are consumed by the ETC to produce ATP. Excess of reducing equivalents leads to reductive stress and this is potentially dangerous, as these compounds harbor high-energy electrons which are likely to initiate chemical reactions with the nearest available oxidants (electron acceptors). Reductive stress is probably not only more common than oxidative stress, but it is also the main source of reactive oxygen species (ROS) [27].

Even though cooling is the most common preservation method, it remains unclear which temperature setting is preferable for optimal organ preservation. Additionally, there is no consensus on what the optimal temperature is for different organs. Thus, the medical community is in need of novel insights to better understand molecular mechanisms of hypothermia. The crux of the problem of choosing the optimal temperature for achieving the maximal benefits in intracellular metabolic status, functionality, and viability of graft lies in weighing the benefits and shortcomings associated with different temperatures. We propose here that the widely accepted view that the therapeutic effect of hypothermia is due to metabolic depression is most likely incomplete. Previous studies have reported that the therapeutic effect of hypothermia peaks at around 32 °C in mammals and no clear benefits arise from decreasing the temperature further [28,29,30,31,32,33]. To the best of our knowledge, no attempts have been made to uncover a mechanistic explanation as to why mild hypothermia (32 °C) is therapeutically more efficient than moderate (28 °C) and deep (22 °C) hypothermia. The main idea of this study was to assess the effect of hypothermia on the activation of cellular stress pathways (the activating component) and the induction of metabolic suppression (the inhibitory component) at various temperatures. To address this issue in the present study, temperatures 37, 32, 28, and 22 °C were chosen to represent normothermia, mild-, moderate-, and deep hypothermia, respectively. Hypoxia and various temperatures were applied in a Latin square design. Cultured human kidney cells (HKC8 cell line) were chosen as a model of hypoxia-sensitive tissue, since kidney transplant is one of the most common organ transplant surgeries performed today. HKC8 cells were subjected to various assays related to the hypoxia response and mechanisms known to mitigate the dangers posed by excess reducing equivalents. We demonstrate that the effects of hypothermia are likely to stem both from metabolic suppression (inhibitory component) and augmentation of stress tolerance (activating component). Metabolic suppression is always enhanced by lowering temperature further (a thermodynamic effect), while the activating component (activation of mechanisms responsible for redox stress tolerance) is not. Together, we suppose that temperatures around 32 °C provide the highest efficacy for the protective effects of hypothermia on the cellular level by supporting both the activating and inhibitory components associated with tissue cooling.

## 2. Results

**Figure 1:**
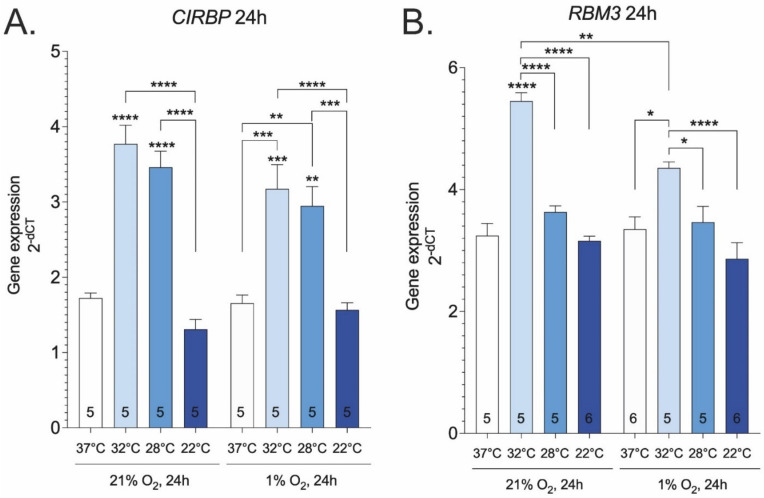
Cirbp (**A**) and Rbm3 (**B**) gene expressions at various temperatures and oxygen concentrations. Asterisks refer to a statistically significant difference with respect to 37 °C control unless indicated otherwise. Numbers in bars indicate sample size. Values are expressed as mean ± SEM. Statistical analysis was performed with One-way ANOVA with post-hoc Tukey HSD Test. *, *p* < 0.05; **, *p* < 0.01; ***, *p* < 0.001; ****, *p* < 0.0001.

As the most basic test of temperature sensitivity, the temperature-dependent induction of well-known hypothermia responsive genes Cirbp (Cold-inducible RNA-binding protein) and Rbm3 (RNA binding motif protein 3) was measured. The induction of cold-inducible genes was most prominent at 32 °C, and further decrease in temperature gradually abolished this effect (Figure 1A,B). Hypoxia interfered with the temperature-dependent induction of Rbm3, whereas it had no such effect on Cirbp. Taken together, these results indicate that the range of temperature sensitivity for Cirbp and Rbm3 is rather narrow, with induction peaking at around 32 °C.

**Figure 2:**
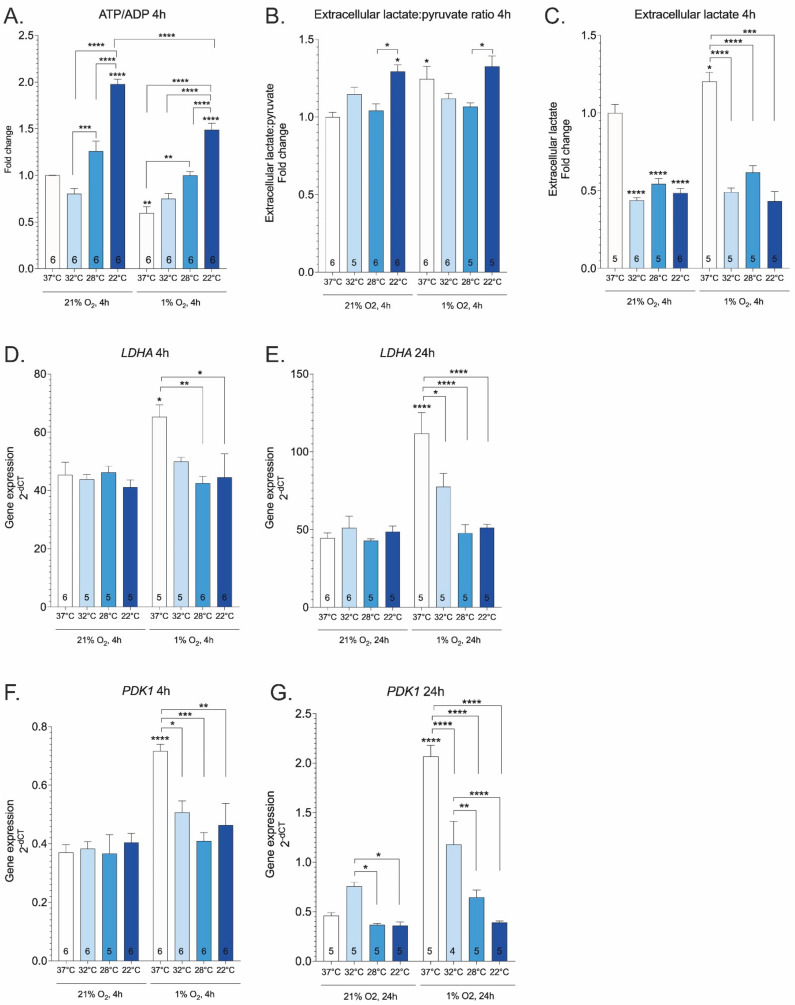
Ratio of ATP to ADP (**A**), ratio of extracellular lactate to pyruvate (**B**), extracellular lactate levels (**C**), and gene expression of LDH-A (**D**,**E**) and PDK1 (**F**,**G**) at various temperatures and oxygen concentrations. Treatments were performed for 4 or 24 h. Results are normalized relative to 37 °C group (**A**–**C**). Asterisks refer to a statistically significant difference with respect to 37 °C control unless indicated otherwise. Numbers in bars indicate sample size. Values are expressed as mean ± SEM. Statistical analysis was performed with One-way ANOVA with post-hoc Tukey HSD Test. *, *p* < 0.05; **, *p* < 0.01; ***, *p* < 0.001; ****, *p* < 0.0001.

The lack of mitochondria-derived ATP has been described as the major driver of pathology in ischemic injury. The most well-known therapeutic mechanism of hypothermia is the preservation of ATP [21,34,35]. As expected, hypoxia reduced ATP/ADP ratio at 37 °C. Hypothermia increased ATP/ADP ratio in both normoxic and hypoxic cells, and this effect was amplified by lowering temperature (Figure 2A). Although prolonged depletion of cellular ATP is expected to promote cell death, emerging data suggest that the accompanying elevation in the NADH:NAD+ ratio can also contribute to pathogenesis [26]. Lactate:pyruvate levels are elevated in hypoxia in response to an increased intracellular NADH:NAD+ ratio [36]. As shown in Figure 2B,C, normothermic hypoxic cells exhibited elevation in the medium lactate:pyruvate ratio and extracellular lactate levels compared to control cells. Hypothermia lowered extracellular lactate levels compared to normothermic incubation (Figure 2C). When 22 °C was applied in isolation or together with hypoxia, extracellular lactate:pyruvate ratio was increased, however, mild and moderate hypothermia (32 °C and 28 °C, respectively) blocked the hypoxia-dependent increase of the extracellular lactate:pyruvate ratio (Figure 2B). In the cytosol, lactate dehydrogenase (LDH) reduces pyruvate to lactate coupled with the oxidation of NADH to NAD+ [37,38]. In response to hypoxia, the expression of LDH is increased (as shown in [39,40,41] and Figure 2D,E). Hypothermia revealed no activation of LDH-A in normoxic cells. In contrast, hypothermia abolished hypoxia-dependent induction of LDH-A gene expression (Figure 2D,E). Hypoxic activation of HIF1-regulated PDK (pyruvate dehydrogenase (PDH) kinase) inhibits PDH in response to elevated NADH and acetyl-CoA [42,43]. Hypothermia significantly reduced PDK1 expression in hypoxic cells (Figure 2F,G). These experiments demonstrate that hypothermia abolishes hypoxia-induced increase in the extracellular lactate:pyruvate ratio, LDH-A, and PDK1 gene expressions that are indicative of reductive stress.

**Figure 3:**
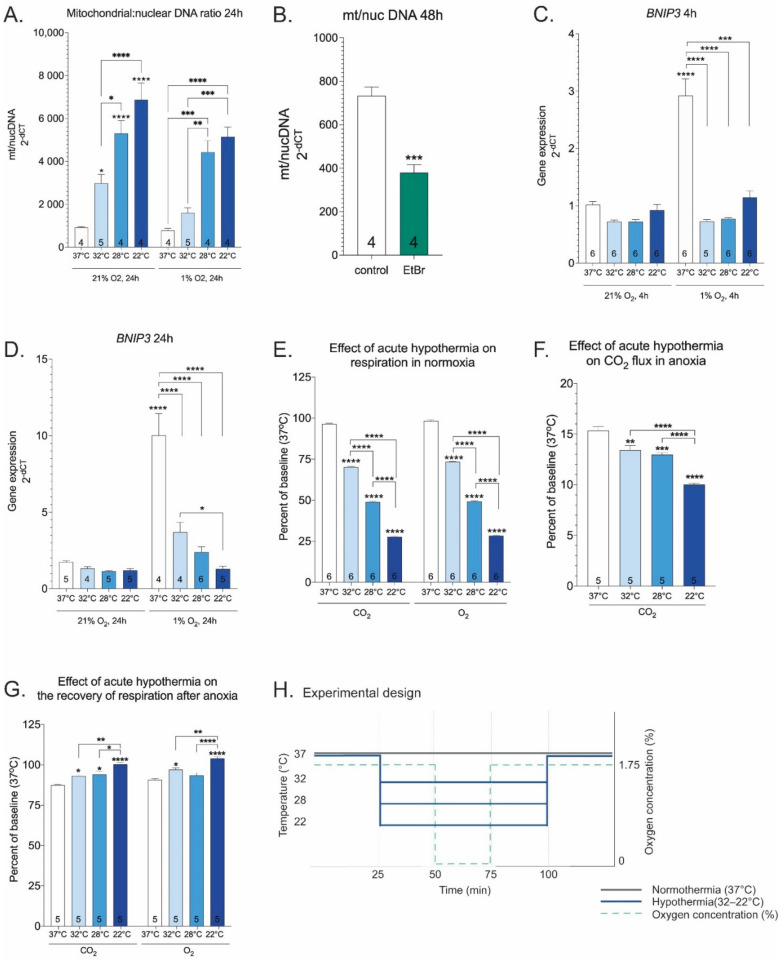
Ratio of mitochondrial DNA to nuclear DNA at various temperatures and oxygen concentrations at 24 h time point (**A**). EtBr (50 ng/mL, 48 h) was used as a positive control to reduce mitochondrial DNA (**B**). BNIP3 gene expression at various temperatures and oxygen concentrations (**C**,**D**). Treatments were performed for 4 or 24 h. Effect of acute hypothermia on respiration in normoxia (**E**). Effect of acute hypothermia on CO_2_ flux in anoxia (**F**). Effect of acute hypothermia on the recovery of respiration after anoxia (**G**). Experiment design (**H**). Asterisks refer to a statistically significant difference with respect to 37 °C control unless indicated otherwise. Numbers in bars indicate sample size. Values are expressed as mean ± SEM. Statistical analysis was performed with One-way ANOVA with post-hoc Tukey HSD Test or unpaired *t* test with Welch correction (**B**). *, *p* < 0.05; **, *p* < 0.01; ***, *p* < 0.001; ****, *p* < 0.0001.

Given that reductive stress causes mitochondrial dysfunction [44,45,46], we sought to determine if hypothermia could influence mitochondrial content and functional phenotype. It was found that hypothermia increases mitochondrial to nuclear DNA ratio monotonically both in isolation and together with hypoxia (Figure 3A). The assay was validated on cells treated with ethidium bromide (EtBr) that is known to interfere with the replication of mitochondrial DNA (Figure 3B). The ratio of mitochondrial DNA to nuclear DNA correlated positively with ATP/ADP ratio (Pearson Product-Moment correlation r = 0.871, *p*-value < 0.01). Bnip3, a canonical HIF1 target gene and a regulator of mitochondrial dysfunction and mitophagy [47], expression was remarkably increased in hypoxia and this effect was quenched by hypothermia (Figure 3C,D). Next, the effect of hypothermia on cellular respiration, the primary indicator of mitochondrial activity, was studied. Mild hypothermia reduced O_2_ and CO_2_ fluxes approximately 25% in normoxia and this effect was amplified by lowering temperature (Figure 3E). Furthermore, hypothermia reduced CO_2_ flux in anoxia, and the effect was amplified by lowering temperature (Figure 3F), although not monotonically. The recovery of respiration was significantly better when anoxic incubation was performed in hypothermic rather than normothermic settings (Figure 3G, see Figure 3H and Figure S1A for experimental setup). In summary, these results suggest that hypothermia influences mitochondrial content, and hypothermia introduced during anoxia enhances the recovery of cellular respiration.

**Figure 4:**
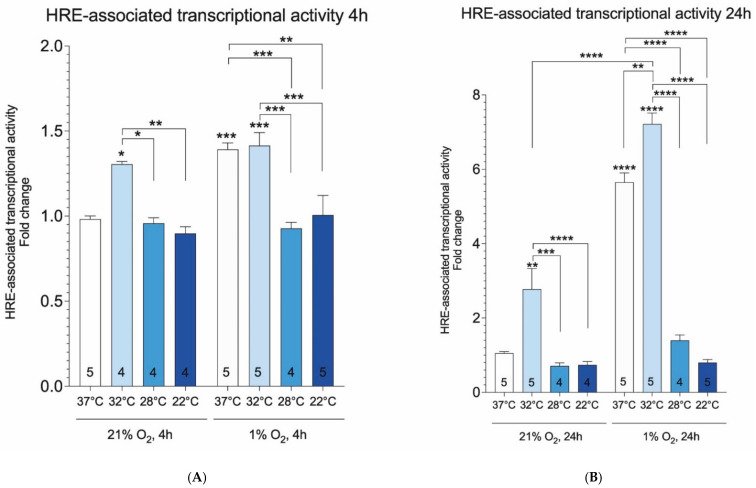
Transcriptional activity of HRE luciferase reporter at various temperatures and oxygen concentrations (**A**,**B**). Treatments were performed for 4 and 24 h. Results are normalized relative to 37 °C group. Asterisks refer to a statistically significant difference with respect to 37 °C control unless indicated otherwise. Numbers in bars indicate sample size. Values are expressed as mean ± SEM. Statistical analysis was performed with One-way ANOVA with post-hoc Tukey HSD Test. *, *p* < 0.05; **, *p* < 0.01; ***, *p* < 0.001; ****, *p* < 0.0001.

Given that HIF1 (hypoxia-inducible factor 1) is the master regulator of hypoxia signaling pathway [48,49,50,51], we sought to determine if there was a direct relationship between hypothermia treatment and HIF1 activation. HIF1 activity was measured based on hypoxia response element (HRE) driven luciferase reporter. Validity of the assay was demonstrated by the induction of HRE reporter by hypoxia after 4 and 24 h at 37 °C. Moderate and deep hypothermia (28 °C and 22 °C, respectively) blocked the hypoxia-dependent induction of HRE reporter (Figure 4A,B). Mild hypothermia (32 °C) induced HRE in normoxic cells and did not interfere with HRE induction in hypoxic cells. These results demonstrate that, in contrast to mild hypothermia, moderate and deep hypothermia interfere with hypoxia-dependent HRE induction.

**Figure 5:**
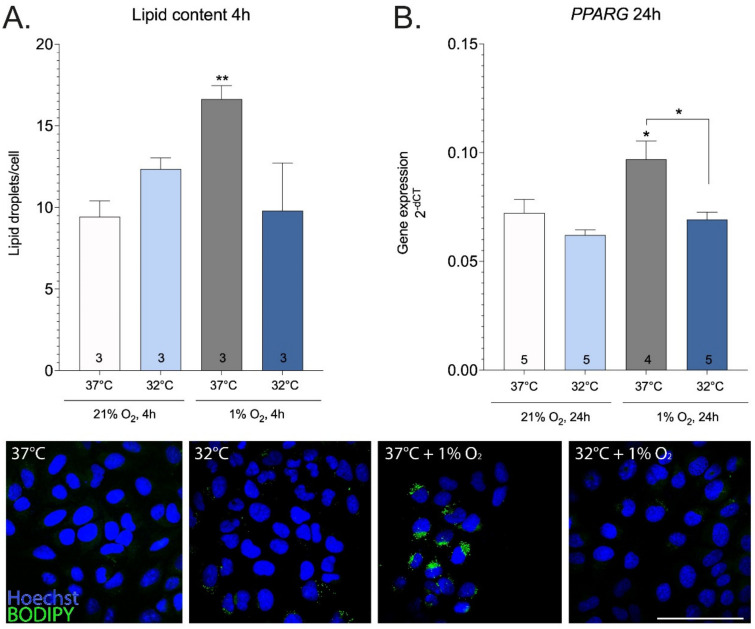
Lipid content (**A**) and PPARG gene expression (**B**) under mild hypothermia (32 °C) and 1% O_2_. Treatments were performed for 4 or 24 h. Scale bar = 100 μm. Asterisks refer to a statistically significant difference with respect to corresponding genotype at 37°C unless indicated otherwise. Numbers in bars indicate sample size. Values are expressed as mean ± SEM. Statistical analysis was performed with unpaired *t* test with Welch correction (**A**) or One-way ANOVA with post-hoc Tukey HSD Test (**B**). *, *p* < 0.05; **, *p* < 0.01.

Another mechanism for mitigating the dangers posed by excess reducing equivalents is the channeling of acetyl-CoA into lipid droplets, which are chemically more stable depos of reductive power. As expected, lipid content was increased by hypoxia as evidenced by increased accumulation of neutral lipid dye BODIPY in lipid droplets (Figure 5A). However, mild hypothermia (32 °C) prevented hypoxia-dependent increase in lipid content. Furthermore, hypoxia-dependent increase in PPARg (peroxisome proliferator- activated receptor gamma) gene expression (fatty acid uptake) was abolished by hypothermia (Figure 5B). Taken together, mild hypothermia counteracts the hypoxia-dependent accumulation of lipid droplets.

## 3. Discussion

During SCS, most of the enzymatic activity is arrested, leading to a dramatic reduction of metabolism [1]. The protective mechanisms of subnormothermia (20–34 °C) have received much less attention. The current study provides a deeper understanding of the mechanisms that could be manipulated in order to ameliorate the consequences of ischemia/reoxygenation. The key novel finding in the present study is that mild hypothermia (32 °C) appears to be optimal for supporting the therapeutic effects of hypothermia, as it supports both the activating and inhibitory components of the response. Importantly, we show that in contrast to mild hypothermia, moderate and deep hypothermia interfere with HIF1-dependent HRE induction in hypoxia. In addition, the present study highlights the potential of reductive stress to provide a common explanation for the key symptoms of ischemia reperfusion injury when compared to the free radical theory.

Our experiments indicate that the expression levels of cold-inducible proteins CIRBP and RBM3 reach their peak at 32 °C in cell culture. CIRBP and RBM3 have been shown to respond to temperature change within a small range [52,53,54] and inhibit apoptosis in vitro [55,56,57], which suggests that the window of mild hypothermia (32 °C) is optimal for achieving the therapeutic effects of CIRBP and RBM3.

ATP depletion is accelerated at temperatures above 30 °C [58], yet cooling to 35 °C remains protective, suggesting that the therapeutic effects of hypothermia involve additional mechanisms besides energy preservation [59,60]. Here, we show that decrements in temperature monotonically increased ATP/ADP ratio. Recent evidence suggests that markedly increased reducing power (i.e., the accumulation of reducing equivalents) is the root cause of ischemia reperfusion injury facilitating oxidative stress and cellular damage [26]. The prototypical causal chain of reductive stress induced reoxygenation injury can be summarized by the following sequence: lack of oxygen → buildup of reducing power → reoxygenation → rapid utilization of accumulated reducing power → burst of ROS (e.g., via reverse electron transport from complex II to complex I). The extracellular lactate:pyruvate ratio has been used as a marker of intracellular NADH:NAD+ status [36] due to the LDH reaction and transport of lactate and pyruvate across the plasma membrane [61]. Lactate:pyruvate levels are often elevated in hypoxic conditions, in response to an increased intracellular NADH:NAD+ ratio. In accordance with previous reports [39,40,41,42,43,62,63], we show that hypoxia increases lactate:pyruvate ratio, LDH-A and PDK1 gene expression. Our experiments with hypothermia revealed that 32 °C and 28 °C decrease the extracellular lactate:pyruvate ratio when applied together with hypoxia, possibly lowering the intracellular NADH:NAD+ ratio in cultured cells. Hypothermia decreased hypoxia-induced expression levels of LDH-A and PDK1. The induction of latter genes shunts pyruvate away from the mitochondria, thereby reducing delivery of acetyl-CoA to CAC, and subsequent consumption of O_2_ in ETC [64].

Importantly, redox couples also link the cellular redox environment with cellular energetics. For instance, NAD+ serves as an electron acceptor pool to support glycolysis, NADH provides electrons for mitochondrial ETC, and NADPH is an electron source for biosynthesis of fatty acids [65]. Mitochondrial CAC and ETC are central to a cell’s metabolism. Both molecular oxygen and reductive potential produced by nutrient breakdown are required for ATP synthesis by ETC. Simultaneous monitoring of the steady state of CAC and ETC in intact cells has proved challenging. We have recently developed an on-line cellular respiration monitoring system to determine O_2_ and CO_2_ fluxes in intact cells in real time. When compared to state-of-the-art technology for monitoring respiration, e.g., the Oroboros 2k and Seahorse XF analyzer, our device has two unique advantages for the present purpose. First, it measures both O_2_ and CO_2_ fluxes directly and simultaneously (Oroboros 2k is only capable of measuring O_2_ flux while Seahorse XF additionally measures extracellular acidification which is a function of glycolytic flux and respiratory activity), giving us a simultaneous readout of the production (CO_2_ flux, mostly contributed by CAC) and O_2_-dependent consumption rates of reducing power (O_2_ flux, mostly ETC activity). Second, the system enables precise and fast control of gas concentrations in the measurement chamber, enabling us to use the normoxia → hypoxia → reoxygenation cycle as a model situation for investigating the clinically relevant hypoxia/reoxygenation scenario. It is well known that the metabolic rate of tissues is positively correlated with body temperature [66,67]. We refer to the notion that the reduction of metabolic rate is responsible for the therapeutic effects of hypothermia as the “thermodynamic hypothesis of therapeutic hypothermia”. In line with the thermodynamic hypothesis, our results show that hypothermia reduces CAC and ETC activities in a coordinated manner. This response can be described with the Arrhenius equation. However, the effect of hypothermia on anoxia CO_2_ seems to be an exception, as it does not follow monotonic decrease. Interestingly, mild hypothermia introduced during anoxia enhances the recovery of respiration after termination of anoxia and hypothermia. The recovery cannot be a thermodynamic effect since it is obtained at 37 °C, suggesting, once again, that hypothermia augments stress tolerance via numerous mechanisms.

Abnormally increased electron pressure leads to mitochondrial dysfunction [68]. We observed a robust increase in mitochondrial content in response to hypothermia in hypoxic cells and it is paralleled by the decreased expression of BNIP3, a mediator of mitophagy signaling. Hypoxic cells have to deal with the excess of reducing equivalents (i.e., molecules carrying high-energy electrons) and this process is a critical step for preventing the transition from reductive stress to cell damage. Accordingly, there have evolved cellular mechanisms to mitigate the dangers posed by reductive equivalents. One of these is the transport of citrate from the mitochondria to cytoplasm, where it is converted to fatty acids and stored as lipid droplets [69], which are chemically more stable depos of reductive power. In the present study, mild hypothermia (32 °C) prevented hypoxia-dependent accumulation of lipid droplets and decreased PPARG gene expression. There is also some evidence that cold temperature can activate brown adipose tissue [70]. As such, it is possible that cold-induced brown adipose tissue activation is another mechanism to provide protection against ischemic conditions. Whether or not this can occur in the setting of ischemia reperfusion injury is unknown. Another mechanism for protecting against the harmful effects of reducing equivalents is the antioxidant system, which seeks to neutralize ROS [71]. In a recent study, our group has demonstrated for the first time that 32 °C activates Nrf2 (nuclear factor erythroid 2-related factor 2), a major regulator of antioxidant gene transcription, in normoxic cells, and provides protection from oxidative stress, presumably by orchestrating adaptive responses to redox stress [23]. Furthermore, our work demonstrated that total glutathione, composed of reduced (GSH) and oxidized forms (GSSG), was significantly increased by hypothermia. Importantly, incubation at 32 °C had no effect on GSSG (glutathione disulfide), suggesting that hypothermia does not lead to elevated reductive stress [23]. However, the downside of the antioxidant system is that it does not deal with the source of the problem (i.e., excess reductive power). Instead of using antioxidants to mitigate oxidative damage, disposal of excessive reducing equivalents should be the main focus. The main drawback of antioxidant compounds is that they often fail to exert therapeutic effects in human studies, presumably because they do not reach the mitochondria where ROS is generated [72]. No additional gain in Nrf2 activity and corresponding target gene expression of the glutathione and thioredoxin system was seen at temperatures below 32 °C [23]. In line with these observations, we show here that only mild hypothermia (32 °C) was sufficient to activate HRE with additional synergistic effect when combined with hypoxia. Whether the observed effect of hypothermia on HRE activation is protective against hypoxic damage is an open question, since hypothermia prevented hypoxia-dependent increase of LDH-A, PDK1, and BNIP3, which are known downstream targets of HIF1.

In summary, the present study explores molecular mechanisms of hypothermia that could be manipulated in order to slow down the negative consequences of hypoxia. It suggests that hypothermia alleviates reductive stress, a seriously understudied phenomenon at the roots of ischemia reperfusion injury. More importantly, the reductive stress hypothesis bears the promise of opening up a new view on potential treatment strategies. The current results are of significant value for translational studies, which are instructed to focus on both the inhibitory and activating effects of therapeutic hypothermia. The present evidence for the effectiveness of hypothermia on alleviating reductive stress applies to mammalian cell culture. These observations need to be corroborated in animal studies before their relevance to ischemia/reperfusion injury can be established.

## 4. Materials and Methods

### 4.1. Cell Lines

Human kidney proximal tubular (HKC8) cells were a gift from Professor Sir Peter J. Ratcliffe. HKC8 cells were cultured in low glucose MEM (Capricorn, Ebsdorfergrund, Germany, MEM-STA) supplemented with 10% FBS (Gibco, Waltham, MA, USA, 10270106), 100 U/mL penicillin, and 100 μg/mL streptomycin (Gibco, Waltham, MA, USA, 15140122) at 37 °C in a 5% CO_2_. Incubator temperature was lowered for hypothermia experiments. A modular incubator chamber (Billups-Rothenberg Inc., San Diego, CA, USA, MIC-101) was used for hypoxia experiments. Culture cells were placed in the chamber, a flow meter was attached to the unit, and the chamber was flushed with 20 L of 1% O_2_, 5% CO_2_, 94% N_2_ gas mixture. The chamber was sealed and placed in the incubator with desired temperature.

### 4.2. Treatments

EtBr was dissolved in milli-Q water with a final concentration of 50 ng/mL. Treatment was performed for 48 h.

Cells were subjected to temperature titration curve from 37 °C to 22 °C for 4 or 24 h either under normoxia (21% O_2_) or hypoxia (1% O_2_). Although hypothermia influences multiple aspects of acute, subacute, and chronic stages of ischemia, we chose to study the effects of hypothermia during the acute phase. First, acceptable maximum preservation times for cold ischemia typically fall between 4 to 24 h [10,11,12,13,14,15,16,17]. Secondly, higher incubation times can trigger secondary events like apoptosis and inflammation [34] making it hard to study the root cause of cellular damage.

### 4.3. Luciferase-Reporter Assay

Cells were seeded at a density of 70,000 cells/well in a 12-well plate, grown overnight, and co-transfected with 187.5 ng pGL4.42[luc2P/HRE/Hygro] (Promega, Madison, WI, USA, E4001) and 12.5 ng pRL-TK (Promega, Madison, WI, USA, E2241) luciferase vectors by Effectene Transfection Reagent (Qiagen, Qiagen, MD, USA, 301425). Next day, cells were kept under various temperatures and oxygen concentrations for 4 and 24 h. Cells were lysed in 1x Passive Lysis Buffer (Promega, Madison, WI, USA, E1941) with gentle shaking at RT for 15 min. The activities of firefly and renilla luciferase were measured using the Dual Luciferase Reporter Assay System (Biotium, Fremont, CA, USA, 30081) according to manufacturer’s protocols. Luminescence was measured on a Promega GloMax Multiplus Plate Reader using a 10-s pre-read delay, followed by a 5-s measurement period.

### 4.4. RNA Extraction

Cells were plated at a density of 400,000 cells in a 6-well plate. The next day, cells were kept under various temperatures and oxygen concentrations for 4 and 24 h, followed by extraction of total RNA with TRIzol^®^ reagent (Thermo Fisher Scientific, Waltham, MA, USA, 15596026) according to the manufacturer’s protocol. Reverse transcription was performed using one microgram of total RNA with random hexamers (LGC Biosearch Technologies, Risskov, Denmark) and SuperScript III Reverse Transcriptase (Thermo Fisher Scientific, Waltham, MA, USA, 18080044).

### 4.5. DNA Extraction

Cells were plated at a density of 400,000 cells in a 6-well plate. The next day, cells were kept under various temperatures and oxygen concentrations for 24 h followed by extraction of DNA with 1× PCR buffer (Bioatlas, Tartu, Estonia) containing proteinase K (final concentration 200 μg/mL). Samples were incubated for 1 h at 56 °C, boiled for 20 min at 97 °C centrifuged 14,000× *g* for 5 min, and stored at −20 °C.

### 4.6. Quantitative Real-Time Reverse Transcription PCR

Every reaction was made in four parallel samples to minimize possible errors. All reactions were performed in a final volume of 10 μL. qPCR was carried out using primers obtained from TAG Copenhagen (Table S1) and HOT FIREPol EvaGreen qPCR Supermix (08-36-00001, Solis BioDyne, Tartu, Estonia). Expression level of HPRT was used as an internal reference. For mitochondrial:nuclear DNA ratio measurement, mitochondrial target was the gene for mt-CytB and nuclear target was a region of H4C. Real-time qPCR reactions were run on QuantStudio 12 K Flex Software v.1.2.2 Real-Time PCR System equipment (Applied Biosystems, Waltham, MA, USA) and quantified with the QuantStudio 12 K Flex Software v.1.2.2.

### 4.7. ADP/ATP Ratio Assay Kit

Cells were plated at a density of 10,000 cells in a 96-well luminometer-compatible tissue culture plate. The next day, cells were kept under various temperatures and oxygen concentrations for 4 h. Ratio of ATP to ADP was measured with ADP/ATP Ratio Assay Kit (Sigma-Aldrich, Burlington, VT, USA, MAK135-1KT) according to the manufacturer’s protocol. Luminescence was measured on a Promega GloMax Multiplus Plate Reader.

### 4.8. Metabolomics

Cells were plated at a density of 450,000/well in a 6-well plate. The next day, cells were washed with PBS, exchanged to medium containing 2% FBS, and kept under various temperatures and oxygen concentrations for 4 h. 100 μL of medium was collected and stored at −80 °C. Targeted metabolomics was carried out with selected hydroxyl acids using a previously described method [73]. For analysis of hydroxy acid, 50 μL of the sample was mixed with 30 μL (500 μM [2H4] succinic acid in methanol). The samples were centrifuged for 15 min at 10,000× *g* and 20 μL was analyzed by liquid chromatography-mass spectrometry QTRAP 4500 (AB Sciex, Vaughan, ON, Canada).

### 4.9. Lipid Content

Cells were plated at a density of 25,000/well onto 8-well culture slides (Falcon^®^, New York, NY, USA, 354118). The next day, cells were kept under various temperatures and oxygen concentrations for 4 h. After the treatments, cells were fixed in 4% paraformaldehyde/PBS solution at 36 °C over 15 min. After washing with PBS five times over 5 min, each cell was stained with BODIPY 493/503 dye (1 µM, Invitrogen, Waltham, MA, USA, D3922) in PBS over 10 min and rinsed thrice with PBS over 5 min. Subsequently, nuclei were stained with 5 μg/mL Bisbenzimide H 33258 (Hoechst 33258, Sigma Aldrich, Burlington, VT, USA) in PBS for 5 min, washed with ddH_2_O, and mounted in Fluoromount (Sigma Aldrich, Burlington, VT, USA) mounting media.

### 4.10. Microscopy and Image Analysis

Fluorescent images were obtained with Olympus FV1200MPE (Olympus, Hamburg, Germany) laser scanning confocal microscope (objective lens: Olympus^TM^ 60X Oil objective, PlanApo, 1.42NA/0.15WD). Near Violet Laser Diode (LD405, 50 mW, Olympus); Sepia Laser Diode (LD473, 15 mW, Olympus) lasers were used in combination with U-MNU2 and UMWIBA3 filter cubes (Olympus) for fluorescence detection. From each replica, three non-overlapping fields of view (211.97 μm × 211.97 μm) were acquired. Images were analyzed using Fiji software [74]. Lipid droplets were counted using the “Find Maxima” tool (prominence > 15). Cell nuclei (cell count) were counted with the “Analyze Particles” tool (images converted to 8 bit format, threshold values were set 1–255, and analyzed particle size: 100-Infinity μm^2^).

### 4.11. Cell Respiration Experiments

We have developed a novel gas flux measurement system to determine O_2_ and CO_2_ fluxes in adherent cell cultures in real time. It allows us to measure the production (by CAC) and utilization (by ETC) of reducing power.

Cells were plated at a density of 8 × 10^6^/100 mm dish on the sterile glass disc (83 mm diameter and 1.15 mm thick glass (Pilkington Microwhite) and grown overnight at 37 °C in a 5% CO_2_. A glass disc was covered with gelatin and placed in the bottom of the 100 mm Petri dish. The next day, cells were washed with 1x PBS, MEM was changed to bicarbonate-free DMEM (Sigma-Aldrich, Burlington, VT, USA, D5030) buffered with 20 mM HEPES, and supplemented with 5.5 mM glucose and 4 mM glutamine. Cells were placed at 37 °C in 0% CO_2_ for 1 h. Before the respiration experiment, carbonic anhydrase (final concentration of 25 μg/mL) (Sigma-Aldrich, Burlington, VT, USA, C2624) was added to the cell culture medium. Optical absorption measurements were performed before and after the respiration experiment. Respiration was normalized to cell number based on cyt-c absorbance (see methods: optical absorption measurements).

Next, the medium was poured off and the disc was placed in the respiration chamber. To prevent the cells from drying out, 0.5 mL medium was pipetted on the disc. The chamber was closed and the system was switched to high throughput mode for 15 s to equilibrate the gasses in the system.

To change gas mix from normoxia to anoxia and vice versa, the system was programmed to follow the next steps: switch for high throughput mode, 14 s for measurement chamber, then switched to reference chamber for 8 s and then switched to measurement chamber for 8 s, after which the system switched back to low throughput mode.

After inserting the disc, O_2_ absorption and CO_2_ emission were measured for 25 min or until the signal stabilized in 37 °C normoxia (~1.7% O_2_, 450–500 ppm CO_2_, 98.2% N_2_), followed by measurement at hypothermic (32, 28 or 22 °C) normoxia for 25 min. After hypothermic normoxia, gas flow was switched to hypothermic anoxia (~0% O_2_, 450–500 ppm CO_2_, 99.9% N_2_) for 25 min, and returned back to 32 °C normoxia for 25 min followed by measurement at 37 °C normoxia for 25 min.

To validate CO_2_ emission, CO_2_ flux in different carbon sources was measured (Figure S1D), and association between CO_2_ flux and cell number was estimated with second order polynomial function (r^2^ = 0.97, Figure S1E). A day after plating, cells were washed with PBS and grown (atmospheric oxygen, atmospheric CO_2_ at 37 °C) in bicarbonate-free DMEM (Sigma-Aldrich, Burlington, VT, USA, D5030), buffered with 20 mM HEPES and various carbon sources for 1 h. (1) Complete medium contained 5.5 mM glucose and 4 mM glutamine, (2) glutamine free medium contained only 5.5 mM glucose, and (3) glucose free medium contained only 4 mM glutamine. O_2_ consumption rate determined by cellular gas flux measurement device correlated well (Pearson *r* = 0.98) with OROBOROs O2k oxygraph measurements in liquid phase, a standard respiration measurement device (Figure S2). In line with previous studies [75,76], constitutive HIF overexpression led to decreased respiratory activity, as evidenced by lower CO_2_ and O_2_ fluxes in VHL (Von Hippel Lindau) KO cells when compared to WT HKC8 cells. This correlation justified the chosen ~1.7% O_2_ gas concentration for normoxic respiration. For theoretical considerations, see O_2_ concentrations calculations and Table S2.

### 4.12. Optical Absorption Measurements

For the cellular gas flux measurement device, respiration signal was normalized to cell mass based on optical absorption. Optical absorption was measured with an integrating sphere (92mm diameter, ~98% efficiency factor), which was calibrated with a 1 cm^2^ black square (absolute dark). Sphere was illuminated by two LEDs (emission maxima 410 and 455 nm) covering reference spectra (455… 475 nm) and absorption maxima of cytochromes [77]. Light emission spectra were recorded with CCD spectro-radiometer PS2000 (Ocean Optics, Dunedin, FL, USA) and optical absorption was calculated as a maximum peak at 415–425 nm range equivalent to *x* mm^2^ of black square. Cells were grown on low absorption glass discs (Pilkington Microwhite glass), which had their individual absorption spectra and weights measured beforehand. A glass disc was placed onto the integrating sphere’s equator to record the absorption. To extrapolate absorption coming from the medium, measurements were repeated with 0.5 mL of added medium. Association (R^2^ = 0.99) between optical absorption and cell number was estimated based on cell numbers counted from the glass disc after respiration measurements (Figure S1C).

### 4.13. Gas System for Simultaneous Measurements of Respiratory CO_2_ and O_2_ Exchange

The system is based on the flow-through principle, where the object of interest is enclosed in an airtight measurement chamber ventilated by a stream of gas containing preset concentrations of N_2_, CO_2_ and O_2_. Due to respiration of the cell culture, O_2_ concentration decreases, and CO_2_ concentration increases in the outlet flow compared to the inlet. The respiration rate is calculated as R = *v*(*C_o_* − *C_i_*), where *C* with subscripts denote concentrations, mole/mole^−1^ (fractional, *i* at the inlet and *o* at the outlet), *v* is the gas flow rate, mole s^−1^, and respiration rate R is expressed in mole s^−1^ per whole object in the chamber. The flow rate was held at 27 μmol/s as measured after the CO_2_ detector (for high throughput mode the flow rate was 245 μmol/s). The flow was held constant by regulating pressure valves of the gas cylinders. By multiplying the concentration difference of inlet and outlet gas flow with the flow rate, we get the respiration rate for the object of interest. Cultured cells were distributed across the surface of a glass disc with diameter 83 mm (54.1 cm^2^) and covered by an approximately 0.1 mm layer of culture medium (0.5 mL spread on the disc surface). This set-up minimizes gas-cell diffusional resistance and complications due to solubility of gasses in the culture medium. Principal gas circuit is presented in Figure S3. The gas mixture was prepared in two gas cylinders (10 L volume), equipped with additional gas entrance valves. The necessary O_2_ concentration was prepared by mixing O_2_, CO_2_, and N_2_ from standard 50 L, 200 bar pressure bottles (AGA, Estonia) at the pressure of up to 10 ba. The gas source to feed the system was chosen by the V1 valve (Figure S3). At the outlet of the V1 valve the gas flow was split into two, and the rates determined the capillary resistances R1 and R2 at the typically adjusted pressure of 650 mb on the dual-step pressure reducing valves PR1 and PR2. Through R2, the flow was directed to the reference Cell1 of the dual-cell Zr O_2_ analyzer S-3A/II (AEI Technologies, Naperville, IL, USA), calibrated against the atmospheric O_2_ concentration. Gas flow through R1 was directed to the measurement block. After R1, the V2 valve enables us to bypass the R1 capillary and create a high-throughput mode with higher flow rate, only limited by low resistance capillary R3. This allowed for faster equilibration of gasses in the measurement block after switching from one gas cylinder to another. The chamber-branch flow first entered the humidifier, embedded in a constant temperature water jacket (items incorporated in the water jacket are encircled by a blue line in Figure S3). The metal chamber body incorporated the segments of the cell chamber and reference channel. The humidifier contained 2 ml of water, layered on the bottom, saturating the gas with water vapor at the adjusted temperature of the water jacket. For example, at 37 °C, by volume, 6% of water vapor additionally evaporated into the dry gas mixture. At the outlet of the humidifier, the gas flow was divided with the help of the two capillaries R4 and R5, generating overpressure of 5 mb in the humidifier. One of these gas flows entered, through R4, the adjacent segment—empty reference channel. Via R5, the other branched flow descended into the cell chamber—a cylindrical volume of 83.5 mm diameter and 3 mm height. In the cell chamber, the gas flowed over the flat surface of the glass disc, exiting from the chamber at its opposite side. In respiration measurements, the O_2_ content of the two gas flows—one through the reference channel and the other through the cell chamber—was compared with the resolution of <1 ppm at the background of 10,000 ppm O_2_. Accordingly, pressure and water vapor content of the gas had to be stabilized with stability better than 10^−4^. To ensure the stability of the system a “gas flow switch” was included. The “gas flow switch” was operated by alternately closing outlets of reference and cell chambers to the sampling circuit with the help of V3 valve. When the valve for either chamber was closed the air stayed static in that chamber, and differences in the other chamber were measured. Alternately, closing the V3 valve ensures reliable undisturbed modulation of the gas flow sampled to the analyzers—altering its origin either from the reference or from the cell chambers. In the case of a cell chamber, the activity of living cells accumulated a higher concentration of CO_2_ and lower concentration of O_2_ which resulted in signal peaks after switching from reference chamber to cell chamber (Figure S1B). These peaks were integrated to calculate the mean respiration during reference channel measurements. The sampled gas enters the ice dryer, a stainless-steel tube placed in icy water, where most of the water vapor is condensed. Before entering O_2_ analyzer, the ice-dry (water vapor pressure 6 mb) gas went through an emergency droplet collector to ensure no water could enter the O_2_ analyzer. After passing through Cell2 of the O2 analyzer, the gas went through the measurement cell of the CO_2_ analyzer (Li-7000, LiCor, Lincoln, NE, USA). The CO_2_ analyzer is operated in differential mode, its reference cell connected to a bottle of N_2_ via the rate-limiting capillary. It was absolutely important to ensure constant total pressure and water vapor partial pressure in the gas entering the analyzers. Due to the slow flow rate, the system needed vacuum-tight installation of metal tubing in order to avoid leaks of atmospheric oxygen. The cell chamber was tightened by vacuum grease (Apiezon M grease, M&I Materials, Manchester, UK) between flat sanded metal surfaces.

### 4.14. O_2_ Concentration Calculations

Basic calculations were done to evaluate the theoretical limit where oxygen diffusion can become limiting for cyt-c. First, saturating oxygen concentration in medium (*C*) was calculated using Henry’s law (1) with a constant of 771.65 mmHg/mM (Table S2) [78].

(1) C=P/H

Secondly, using Fick’s first law (2), the maximum thickness of the medium layer to support oxygen diffusion at measured respiratory rate was calculated.

(2) F=D×ΔC/Δx

The rate of diffusion (*F*) was equated to the maximal measured oxygen consumption rate (*F* = 1.05 × 10^−8^ mM/s/cm^2^). Diffusion coefficients (D) of 2.84 × 10^−5^ cm^2^/s (without fetal bovine serum, FBS) and 2.69 × 10^−5^ cm^2^/s (with 10% FBS) at 37 °C were used [78]. Concentration difference (ΔC) in the medium was calculated as a difference between saturated O_2_ concentration at the surface and the concentration for 91% cyt-c saturation at the cell layer (10*x*Km, Km_cyt-c_ = 0.5 × 10^−3^ mM) [79]. Solving for Δx (3), the maximum medium layer thickness supporting measured respiration rate was calculated (Table S2).

(3) Δx=D×ΔC/F

Comparing the actual medium layer (0.1 mm) and the maximum medium layer supporting measured respiration (0.32 mm), we estimate that the oxygen concentration of 1.75% was most likely not limiting the cyt-c activity. For hypoxia (1% O_2_) experiments done in 6-well, the same oxygen consumption rate could inhibit cyt-c activity. Although HIF can have a much higher Km for oxygen, it can take up to 6 h to take full effect on respiration inhibition [43].

### 4.15. Respirometric Analysis with Oroboros

The cellular gas flux measurement system was validated with Oroboros machine using WT and VHL KO HKC8 cells. The cellular routine respiration, reflecting the aerobic metabolic activity in complete medium (Capricorn, Ebsdorfergrund, Germany, MEM-STA), was recorded by high-resolution respirometry (Oroboros Instruments, Innsbruck, Austria) with a Clark electrode at 37 °C. Cells were collected by trypsinization, centrifugation, and titrated to 0.6 × 10^6^ cells/ml. Roughly 1.2 × 10^6^ cells were transferred into each respiratory chamber of the oxygraph. The slopes of O_2_ consumption were calculated with DatLab 4.0 (Oroboros).

### 4.16. Statistical Analysis

Statistical significance of experimental treatments was evaluated as follows: (1) unpaired *t*-test with Welch correction for comparing two samples; and (2) a 1-way ANOVA with a Tukey test as the post hoc analysis for treatments with more than two levels. *p* < 0.05 denoted statistical significance. All statistical analysis was performed using Prism 8 (GraphPad Software Inc., San Diego, CA, USA).

## 5. Conclusions

This study addresses an important question on why hypothermia might be effective in reducing hypoxic tissue damage. Here, we suggest that the efficacy of hypothermia depends on a mechanism with inhibitory (e.g., the slowing down of metabolic rate) and activating (e.g., mechanisms for mitigating the dangers posed by excess reducing equivalents) components with the highest optimal activation of both components emerging in the window of mild hypothermia (32 °C). It suggests that hypothermia could be one of the exogenous mechanisms that could help to ameliorate reductive pressure caused by the shift in the ratio of important redox couples (NADH/NAD+ and NADPH/NAD+). The current evidence highlights potentially therapeutic molecular mechanisms that can be translated to in vivo models for further evaluation of therapeutic efficacy. Mechanistic understanding of the effects of hypothermia is required to make informed decisions on how to enhance the efficacy of clinical hypothermia.

## Data Availability

Not applicable.

## Supplementary Materials

The supporting information can be downloaded at: https://www.mdpi.com/article/10.3390/ijms231710108/s1.

## References

[1] Krezdorn N., Tasigiorgos S., Wo L., Turk M., Lopdrup R., Kiwanuka H., Win T.-S., Bueno E., Pomahac B. (2017). Tissue conservation for transplantation. Innov. Surg. Sci..

[2] Moers C., Pirenne J., Paul A., Ploeg R.J. (2012). Machine Perfusion or Cold Storage in Deceased-Donor Kidney Transplantation. N. Engl. J. Med..

[3] Taylor M.J., Baicu S.C. (2010). Current state of hypothermic machine perfusion preservation of organs: The clinical perspective. Cryobiology.

[4] Brettschneider L., Daloze P.M., Huguet C., Groth C.G., Kashiwagi N., Hutchison D.E., Starzl T.E. (1967). Successful orthotopic transplantation of liver homografts after eight to twenty-five hours preservation. Surg. Forum.

[5] Van Rijn R., Schurink I.J., de Vries Y., Berg A.P.V.D., Cerisuelo M.C., Murad S.D., Erdmann J.I., Gilbo N., de Haas R.J., Heaton N. (2021). Hypothermic Machine Perfusion in Liver Transplantation-A Randomized Trial. N. Engl. J. Med..

[6] Jing L., Yao L., Zhao M., Peng L.-P., Liu M. (2018). Organ preservation: From the past to the future. Acta Pharmacol. Sin..

[7] Zanetto A., Russo F.P., Germani G., Burra P. (2018). Organ Preservation in Liver Transplantation. Semin. Liver Dis..

[8] Vairetti M., Ferrigno A., Carlucci F., Tabucchi A., Rizzo V., Boncompagni E., Neri D., Gringeri E., Freitas I., Cillo U. (2009). Subnormothermic machine perfusion protects steatotic livers against preservation injury: A potential for donor pool increase?. Liver Transplant..

[9] Carrel A., Lindbergh C.A. (1935). The Culture of Whole Organs. Science.

[10] Collins G., Bravo-Shugarman M., Terasaki P. (1969). Kidney preservation for transportation: Initial Perfusion and 30 Hours’ Ice Storage. Lancet.

[11] Jahania M., Sanchez J., Narayan P., Lasley R.D., Mentzer R.M. (1999). Heart preservation for transplantation: Principles and strategies. Ann. Thorac. Surg..

[12] Kosieradzki M., Kuczynska J., Piwowarska J., Wegrowicz-Rebandel I., Kwiatkowski A., Lisik W., Michalak G., Danielewicz R., Paczek L., Rowinski W.A. (2003). Prognostic significance of free radicals: Mediated injury occurring in the kidney donor. Transplantation.

[13] Dhital K.K., Iyer A., Connellan M., Chew H.C., Gao L., Doyle A., Hicks M., Kumarasinghe G., Soto C., Dinale A. (2015). Adult heart transplantation with distant procurement and ex-vivo preservation of donor hearts after circulatory death: A case series. Lancet.

[14] Machuca T.N., Mercier O., Collaud S., Tikkanen J., Krueger T., Yeung J.C., Chen M., Azad S., Singer L., Yasufuku K. (2015). Lung Transplantation with Donation After Circulatory Determination of Death Donors and the Impact of Ex Vivo Lung Perfusion. Am. J. Transplant..

[15] Acceptable Ischemic Times. https://www.nedonation.org/donation-guide/organ/acceptable-ischemic-times.

[16] Monbaliu D., Pirenne J., Talbot D. (2011). Liver transplantation using Donation after Cardiac Death donors. J. Hepatol..

[17] Monbaliu D., Brassil J. (2010). Machine perfusion of the liver: Past, present and future. Curr. Opin. Organ Transplant..

[18] Liu W.-P., Humphries A.L., Russell R., Stoddard L.D., Moretz W.H. (1971). 48-Hour Storage of Canine Kidneys after Brief Perfusion with Collinsʼ Solution. Ann. Surg..

[19] Weissenbacher A., Vrakas G., Nasralla D., Ceresa C.D.L. (2019). The future of organ perfusion and re-conditioning. Transpl. Int..

[20] Ponticelli C.E. (2015). The impact of cold ischemia time on renal transplant outcome. Kidney Int..

[21] Talma N., Kok W., Mestdagh C.D.V., Shanbhag N., Bouma H., Henning R. (2016). Neuroprotective hypothermia-Why keep your head cool during ischemia and reperfusion. Biochim. Biophys. Acta.

[22] Yu S.P., Lee J.H., Zhang J. (2017). Neuroprotective mechanisms and translational potential of therapeutic hypothermia in the treatment of ischemic stroke. Neural Regen. Res..

[23] Eskla K.-L., Porosk R., Reimets R., Visnapuu T., Vasar E., Hundahl C.A., Luuk H. (2018). Hypothermia augments stress response in mammalian cells. Free Radic. Biol. Med..

[24] Tahir R., Pabaney A. (2016). Therapeutic hypothermia and ischemic stroke: A literature review. Surg. Neurol. Int..

[25] Luscombe M., Andrzejowski J.C. (2006). Clinical applications of induced hypothermia. Contin. Educ. Anaesth. Crit. Care Pain.

[26] Chouchani E.T., Pell V.R., Gaude E., Aksentijević D., Sundier S.Y., Robb E.L., Logan A., Nadtochiy S.M., Ord E.N.J., Smith A.C. (2014). Ischaemic accumulation of succinate controls reperfusion injury through mitochondrial ROS. Nature.

[27] Ghyczy M., Boros M. (2002). Evidence in support of a concept of reductive stress-Reply by Ghyczy & Boros. Br. J. Nutr..

[28] Bernard S.A., Gray T.W., Buist M.D., Jones B.M., Silvester W., Gutteridge G., Smith K. (2002). Treatment of Comatose Survivors of Out-of-Hospital Cardiac Arrest with Induced Hypothermia. N. Engl. J. Med..

[29] Hypothermia after Cardiac Arrest Study Group (2002). Mild therapeutic hypothermia to improve the neurologic outcome after cardiac arrest. N. Engl. J. Med..

[30] Dalen M.L., Liu X., Elstad M., Løberg E.M. (2012). Resuscitation with 100% oxygen increases injury and counteracts the neuroprotective effect of therapeutic hypothermia in the neonatal rat. Pediatr. Res..

[31] Bona E., Hagberg H., Løberg E.M., Bågenholm R. (1998). Protective effects of moderate hypothermia after neonatal hypoxia-ischemia: Short- and long-term outcome. Pediatr. Res..

[32] Holzer M. (2010). Targeted Temperature Management for Comatose Survivors of Cardiac Arrest. N Engl. J. Med..

[33] Wood T., Osredkar D., Puchades M., Maes E., Falck M., Flatebø T., Walløe L., Sabir H., Thoresen M. (2016). Treatment temperature and insult severity influence the neuro-protective effects of therapeutic hypothermia. Sci. Rep..

[34] Yenari M.A., Han H.S. (2012). Neuroprotective mechanisms of hypothermia in brain ischaemia. Nat. Rev. Neurosci..

[35] Jassem W., Heaton N.D. (2004). The role of mitochondria in ischemia/reperfusion injury in organ transplantation. Kidney Int..

[36] Williamson D.H., Lund P., Krebs H.A. (1967). The redox state of freenicotinamide-adenine dinucleotide in the cytoplasm and mitochondria of ratliver. Biochem. J..

[37] Xiao W., Wang R.-S., Handy D.E., Loscalzo J. (2018). NAD(H) and NADP(H) Redox Couples and Cellular Energy Metabolism. Antioxid. Redox Signal..

[38] Lunt S.Y., Vander Heiden M.G. (2011). Aerobic Glycolysis: Meeting the Metabolic Requirements of Cell Proliferation. Annu. Rev. Cell Dev. Biol..

[39] Robin E.D., Murphy B.J., Theodore J.J. (1984). Coordinate regulation of glycolysis by hypoxia in mammalian cells. Cell. Physiol..

[40] Marti H.H., Jung H.H., Pfeilschifter J., Bauer C. (1994). Hypoxia and cobalt stimulate lactate dehydrogenase (LDH) activity in vascular smooth muscle cells. Eur. J. Physiol..

[41] Firth J.D., Ebert B.L., Pugh C.W., Ratcliffe P.J. (1994). Oxygen-regulated control elements in the phosphoglycerate kinase 1 and lactate dehydrogenase A genes: Similarities with the erythropoietin 3′ enhancer. Proc. Natl. Acad. Sci. USA.

[42] Kim J.-W., Tchernyshyov I., Semenza G.L., Dang C.V. (2006). HIF-1-mediated expression of pyruvate dehydrogenase kinase: A metabolic switch required for cellular adaptation to hypoxia. Cell Metab..

[43] Papandreou I., Cairns R.A., Fontana L., Lim A.L., Denko N.C. (2006). HIF-1 mediates adaptation to hypoxia by actively downregulating mitochondrial oxygen consumption. Cell Metab..

[44] Lambert A.J., Brand M.D. (2004). Superoxide production by NADH: Ubiquinone oxidoreductase (complex I) depends on the pH gradient across the mitochondrial inner membrane. Biochem. J..

[45] Kussmaul L., Hirst J. (2006). The mechanism of superoxide production by NADH:ubiquinone oxidoreductase (complex I) from bovine heart mitochondria. Proc. Natl. Acad. Sci. USA.

[46] Murphy M.P. (2009). How mitochondria produce reactive oxygen species. Biochem. J..

[47] Zhang J., Ney P.A. (2009). Role of BNIP3 and NIX in cell death, autophagy, and mitophagy. Cell Death Differ..

[48] Manalo D.J., Rowan A., Lavoie T., Natarajan L., Kelly B.D., Ye S.Q., Garcia J.G.N., Semenza G.L. (2005). Transcriptional regulation of vascular endothelial cell responses to hypoxia by HIF-1. Blood.

[49] Elvidge G.P., Glenny L., Appelhoff R.J., Ratcliffe P.J., Ragoussis J., Gleadle J.M. (2006). Concordant Regulation of Gene Expression by Hypoxia and 2-Oxoglutarate-dependent Dioxygenase Inhibition. J. Biol. Chem..

[50] Semenza G.L., Wang G.L. (1992). A nuclear factor induced by hypoxia via de novo protein synthesis binds to the human erythropoietin gene enhancer at a site required for transcriptional activation. Mol. Cell. Biol..

[51] Forsythe J., Jiang B.H., Iyer N.V., Agani F., Leung S.W., Koos R.D., Semenza G.L. (1996). Activation of vascular endothelial growth factor gene transcription by hypoxia-inducible factor 1. Mol. Cell. Biol..

[52] Tong G., Endersfelder S., Rosenthal L.-M., Wollersheim S., Sauer I.M., Bührer C., Berger F., Schmitt K.R.L. (2013). Effects of moderate and deep hypothermia on RNA-binding proteins RBM3 and CIRP expressions in murine hippocampal brain slices. Brain Res..

[53] Rzechorzek N.M., Connick P., Patani R., Selvaraj B.T., Chandran S. (2015). Hypothermic Preconditioning of Human Cortical Neurons Requires Proteostatic Priming. eBioMedicine.

[54] Nishiyama H., Itoh K., Kaneko Y., Kishishita M., Yoshida O., Fujita J. (1997). A Glycine-rich RNA-binding Protein Mediating Cold-inducible Suppression of Mammalian Cell Growth. J. Cell Biol..

[55] Chip S., Zelmer A., Ogunshola O.O., Felderhoff-Mueser U., Nitsch C., Bührer C., Wellmann S. (2011). The RNA-binding protein RBM3 is involved in hypothermia induced neuroprotection. Neurobiol. Dis..

[56] Zhang H.-T., Xue J.-H., Zhang Z.-W., Kong H.-B., Liu A.-J., Li S.-C., Xu D.-G. (2015). Cold-inducible RNA-binding protein inhibits neuron apoptosis through the suppression of mitochondrial apoptosis. Brain Res..

[57] Kita H., Carmichael J., Swartz J., Muro S., Wyttenbach A., Matsubara K., Rubinsztein D.C., Kato K. (2002). Modulation of polyglutamine-induced cell death by genes identified by expression profiling. Hum. Mol. Genet..

[58] Ning X.-H., Xu C.-S., Song Y.C., Childs K.F., Xiao Y., Bolling S.F., Lupinetti F.M., Portman M.A. (1998). Temperature Threshold and Modulation of Energy Metabolism in the Cardioplegic Arrested Rabbit Heart. Cryobiology.

[59] Kanemoto S., Matsubara M., Noma M., Leshnower B.G., Parish L.M., Jackson B.M., Hinmon R., Hamamoto H., Gorman J.H., Gorman R.C. (2009). Mild hypothermia to limit myocardial ischemia-reperfusion injury: Importance of timing. Ann. Thorac. Surg..

[60] Shao Z., Sharp W.W., Wojcik K.R., Li C., Han M., Chang W., Ramachandran S., Li J., Hamann K.J., Hoek T.L.V. (2010). Therapeutic hypothermia cardioprotection via Akt- and nitric oxide-mediated attenuation of mitochondrial oxidants. Am. J. Physiol. Heart Circ. Physiol..

[61] Poole R.C., Halestrap A.P. (1993). Transport of lactate and other monocarboxylatesacross mammalian plasma membranes. Am. J. Physiol..

[62] Hillered L., Vespa P.M., Hovda D.A. (2005). Translational Neurochemical Research in Acute Human Brain Injury: The Current Status and Potential Future for Cerebral Microdialysis. J. Neurotrauma.

[63] Tisdall M.M., Smith M. (2006). Cerebral microdialysis: Research technique or clinical tool. Br. J. Anaesth..

[64] Xiang L., Semenza G.L. (2019). Hypoxia-inducible factors promote breast cancer stem cell specification and maintenance in response to hypoxia or cytotoxic chemotherapy. Adv. Cancer Res..

[65] Ying W. (2008). NAD^+^/NADH and NADP^+^/NADPH in Cellular Functions and Cell Death: Regulation and Biological Consequences. Antioxid. Redox Signal..

[66] Chaui-Berlinck J.G., Monteiro L.H.A., Navas C.A., Bicudo J.E.P.W. (2002). Temperature effects on energy metabolism: A dynamic system analysis. Proc. R. Soc. B Boil. Sci..

[67] Geiser F. (2004). Metabolic Rate and Body Temperature Reduction During Hibernation and Daily Torpor. Annu. Rev. Physiol..

[68] Lipinski B. (2002). Evidence in support of a concept of reductive stress. Br. J. Nutr..

[69] Mylonis I., Simos G., Paraskeva E. (2019). Hypoxia-Inducible Factors and the Regulation of Lipid Metabolism. Cells.

[70] Seki T., Yang Y., Sun X., Lim S., Xie S., Guo Z., Xiong W., Kuroda M., Sakaue H., Hosaka K. (2022). Brown-fat-mediated tumour suppression by cold-altered global metabolism. Nature.

[71] Murphy E., Steenbergen C. (2008). Mechanisms Underlying Acute Protection from Cardiac Ischemia-Reperfusion Injury. Physiol. Rev..

[72] Frei B. (2004). Efficacy of Dietary Antioxidants to Prevent Oxidative Damage and Inhibit Chronic Disease. J. Nutr..

[73] Porosk R., Terasmaa A., Mahlapuu R., Soomets U., Kilk K. (2017). Metabolomics of the Wolfram Syndrome 1 Gene (Wfs1) Deficient Mice. J. Integr. Biol..

[74] Schindelin J., Arganda-Carreras I., Frise E., Kaynig V., Longair M., Pietzsch T., Preibisch S., Rueden C., Saalfeld S., Schmid B. (2012). Fiji: An open-source platform for biological-image analysis. Nat. Methods.

[75] Simonnet H., Alazard N., Pfeiffer K., Gallou C., Beroud C., Demont J., Bouvier R., Schägger H., Godinot C. (2002). Low mitochondrial respiratory chain content correlates with tumor aggressiveness in renal cell carcinoma. Carcinogenesis.

[76] Favier J., Brière J.-J., Burnichon N., Rivière J., Vescovo L., Benit P., Giscos-Douriez I., De Reyniès A., Bertherat J., Badoual C. (2009). The Warburg Effect Is Genetically Determined in Inherited Pheochromocytomas. PLoS ONE.

[77] Moore C.L., Strasberg P.M. (1970). Metabolic Reactions in the Nervous System.

[78] Place T.L., Domann F.E., Case A.J. (2017). Limitations of oxygen delivery to cells in culture: An underappreciated problem in basic and translational research. Free Radical. Bio. Med..

[79] Scandurra F.M., Gnaiger E. (2010). Cell respiration under hypoxia: Facts and artefacts in mitochondrial oxygen kinetics. Adv Exp Med Biol..

