# Supplementary material for: Hypothermia Alleviates Reductive Stress, a Root Cause of Ischemia Reperfusion Injury
Source: Int J Mol Sci. 2022 Sep 3;23(17):10108. doi: 10.3390/ijms231710108 (PMC9456258; doi:10.3390/ijms231710108)
Supplement: Supplementary file 1 [file ijms-23-10108-s001.zip › ijms-1865758-supplementary.pdf]

Supplementary Material

Hypothermia alleviates reductive stress, a root cause of ischemia reperfusion injury

Kattri-Liis Eskla[a, b, 1,*], Hans Vellama[a, b, 1], Liisi Tarve [a,b], Hillar Eichelmann[c,d], Toomas Jagomäe[a,b], Rando Porosk[e], Vello Oja[d, 2], Heikko Rämma[d], Nadežda Peet[c], Agu Laisk[d], Vallo Volke[c], Eero Vasar[a,b], Hendrik Luuk[a,b]

[a] Institute of Biomedicine and Translational Medicine, Department of Physiology, University of Tartu, Tartu, Estonia

[b] Center of Excellence for Genomics and Translational Medicine, University of Tartu, Tartu, Estonia

[c] Institute of Biomedicine and Translational Medicine, Department of Pathophysiology, University of Tartu, Tartu, Estonia

[d] Institute of Technology, University of Tartu, Tartu, Estonia

[e] Institute of Biomedicine and Translational Medicine, Department of Biochemistry, University of Tartu, Tartu, Estonia

[1] These authors contributed equally
[2] Deceased

[*] Corresponding author

Table S1. Primers used for qPCR analysis

Gene name	Forward primer 5' 3'	Reverse primer 5' 3'
BNIP3	GACGCACAGCATGAGTCTGGAC	CTTGACCAATCCCATATCCAATCTGAG
CIRBP	GCCATGGCATCAGATGAAGGC	CTGTCTTTCACAACCACCACTTC
CYT-B	TAGCAATAATCCCCATCCTCCATATATCC	ACTTGTCCAATGATGGTAAAAGGGTAGC
H4C	GCTCCGGGATAACATCCAGGGCATT	CCTGACGTTTTAGGGCATATACTACATC
HPRT1	GACTTTGCTTTCCTTGGTCAGG	AGTCTGGCTTATATCCAACACTTCG
LDHA	GCTGGGAGTTCACCCATTAAGCTG	CAATAGCCAGGATGTGTAGCCTTTGAG
PDK1	ATCAGTGAATGCTTGTGAAAAGACCTC	CTGAAGACTCTGGATATACCAGCTTTGTA
PPARG	AGCCTGCATCTCCACCTTATTATTCTGAG	GCTTCAATCTGATTGTTCTCCGGAAGAAAC
RBM3	CTCTGAAGAAGGAAAGCTCTTCG	GTCCTTGACAACGACCACCTC

Table S2. O_2 concentrations calculations

Experiment environment (cm^2)	O_2 partial pressure in gas (mmHg)	O_2 concentration in medium (mM)	Diffusion coefficient - D (cm^2/s)	ΔC (mM)	Maximum medium layer thickness Δx (mm)	Actual medium layer (mm)
Hypoxic 6-well S = 9.62	7.6 (1% O_2)	0.0099	2.69×10^{-5}	0.0049	~0.13	~2.1
Normoxic Petri dish S = 58.1	141.4 (18.6% O_2)	0.181	2.69×10^{-5}	0.176	~4.51	~1.7
Normoxic Glass plate S = 54.11	13.3 (1.75% O_2)	0.0173	2.84×10^{-5}	0.0123	~0.33	~0.1

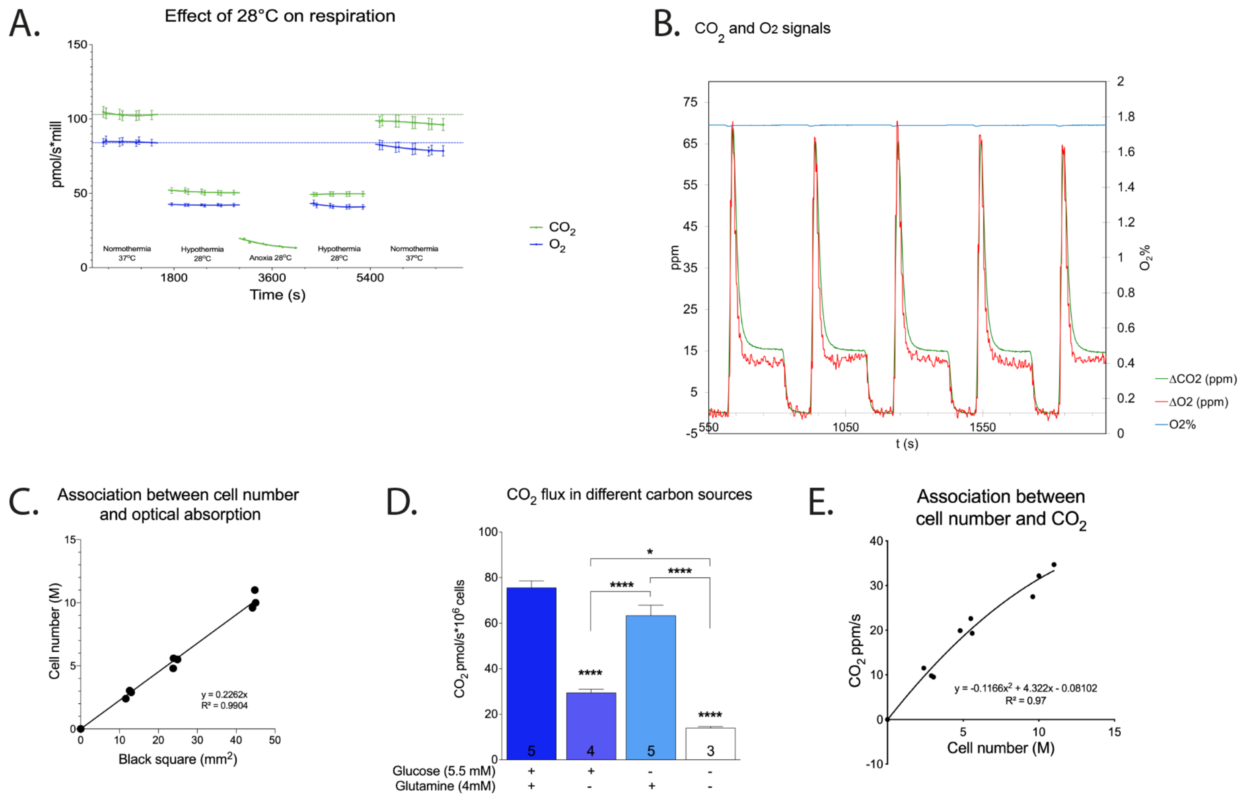

Figure S1. Example of experiment set-up showing effect of 28°C on respiration as measured by the cellular gas flux measurement device (A). CO_2 and O_2 signals as measured by the cellular gas flux measurement device (B). Measurements were recorded by switching between the reference chamber (100 sec) and the cell chamber (200 sec). Association between cell number and optical absorption (C). CO_2 flux in different carbon sources (D). Association between CO_2 flux and cell number (E). HKC-8 cells were used in all experiments. The asterisks refer to a statistically significant difference with respect to the complete medium unless indicated otherwise. Number on the bar indicates sample size. Statistical analysis was performed with One-way ANOVA with post-hoc Tukey HSD Test. *, $p < 0.05$; ****, $p < 0.0001$.

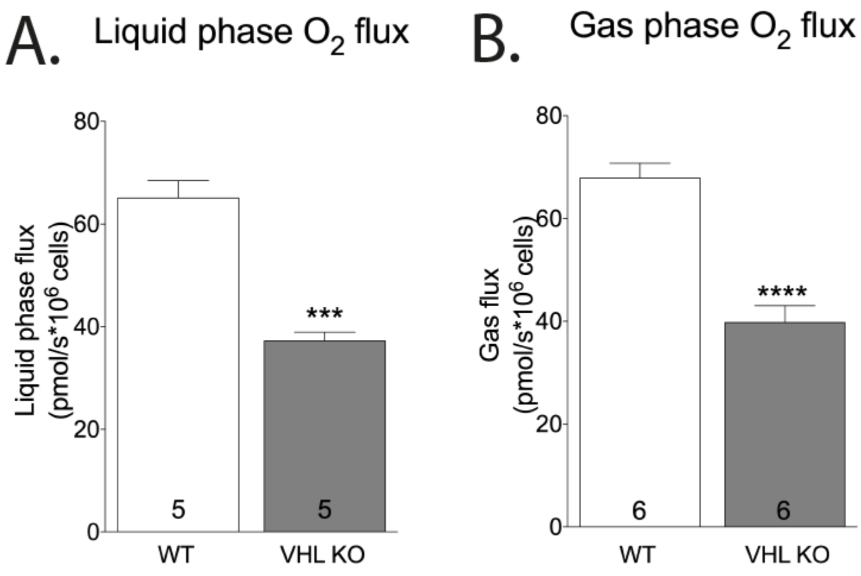

Figure S2. Genotype effect on O_2 flux as measured by Oroboros oxygraph in liquid phase (A) and O_2 flux as measured by cellular gas flux measurement device (B). The asterisks refer to a statistically significant difference with respect to the WT cells unless indicated otherwise. Number on the bar indicates sample size. Values are expressed as mean ± SEM. Statistical analysis was performed with unpaired t-test with Welch's correction. *** $p < 0.001$; ****, $p < 0.0001$

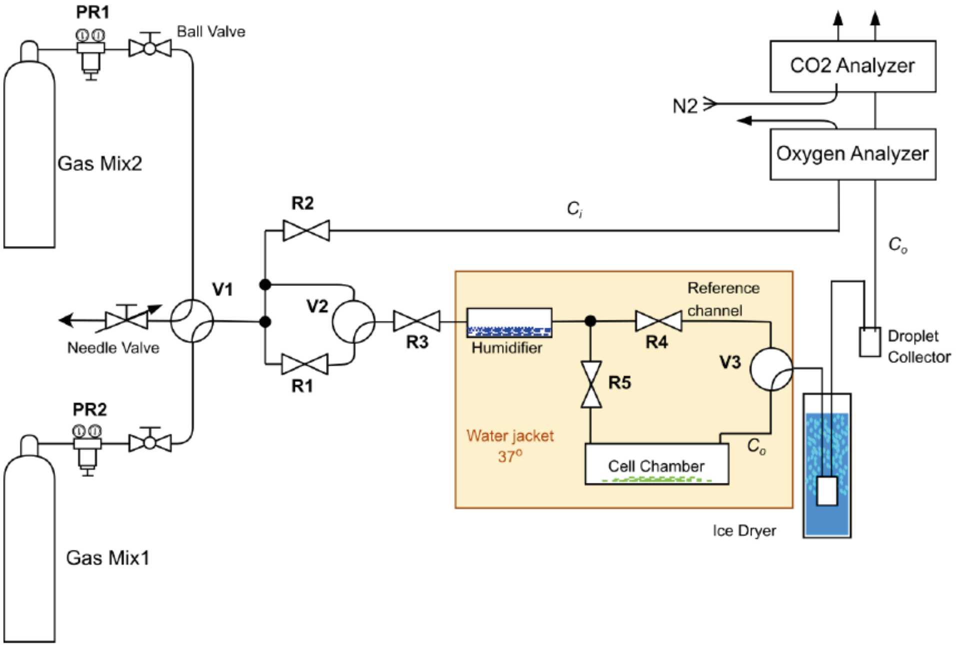

Figure S3. Diagram of the off-gas measurement device. Respiration rate was calculated from the difference between outlet gas concentration (C_o) and inlet gas concentration (C_i), from $R=v(C_o-C_i)$ where v is the gas flow rate, mole*s^{-1}, and R is respiration rate expressed in mole*s^{-1} per whole object in the chamber. PR1, PR2 - pressure reducing valves; V1, V2, V3 - automated valves; R1, R2, R3, R4, R5 - resistances capillaries.
